# p53-R273H Sustains ROS, Pro-Inflammatory Cytokine Release and mTOR Activation While Reducing Autophagy, Mitophagy and UCP2 Expression, Effects Prevented by wtp53

**DOI:** 10.3390/biom11030344

**Published:** 2021-02-24

**Authors:** Maria Anele Romeo, Maria Saveria Gilardini Montani, Rossella Benedetti, Andrea Arena, Gabriella D’Orazi, Mara Cirone

**Affiliations:** 1 Department of Experimental Medicine, Sapienza University of Rome, 00161 Rome, Italy; mariaanele.romeo@uniroma1.it (M.A.R.); mariasaveria.gilardinimontani@uniroma1.it (M.S.G.M.); benedetti.1589832@studenti.uniroma1.it (R.B.); aarena026@gmail.com (A.A.); 2 Laboratory Affiliated to Istituto Pasteur Italia-Fondazione Cenci Bolognetti, 00161 Rome, Italy; 3 Department of Research, IRCCS Regina Elena National Cancer Institute, 00144 Rome, Italy; gdorazi@unich.it; 4 Department of Neurosciences, Images and Clinical Sciences, University “G. d’Annunzio”, 66013 Chieti, Italy

**Keywords:** mutp53, wtp53, ROS, cytokines, cancer

## Abstract

*p53* is the most frequently mutated or inactivated gene in cancer, as its activity is not reconcilable with tumor onset and progression. Moreover, mutations in the *p53* gene give rise to mutant proteins such as p53-R273H that, besides losing the wild type p53 (wtp53) capacity to safeguard genome integrity, may promote carcinogenesis, mainly due to its crosstalk with pro-oncogenic pathways. Interestingly, the activation of oncogenic pathways is interconnected with reactive oxygen species (ROS) and the release of pro-inflammatory cytokines that contribute to create an inflammatory/pro-tumorigenic milieu. In this study, based on experiments involving p53-R273H silencing and transfection, we showed that this mutant p53 (mutp53) promoted cancer cell survival by increasing intracellular ROS level and pro-inflammatory/immune suppressive cytokine release, activating mTOR, reducing autophagy and mitophagy and downregulating uncoupling protein 2 (UCP2). Interestingly, p53-R273H transfection into cancer cells carrying wtp53 induced none of these effects and resulted in p21 upregulation. This suggests that wtp53 may counteract several pro-tumorigenic activities of p53-R273H and this could explain the lower aggressiveness of cancers carrying heterozygous mutp53 in comparison to those harboring homozygous mutp53.

## 1. Introduction

Several p53 mutant proteins (mutp53), particularly the contact mutant proteins such as p53-R273H, may act as oncogenes, a behavior that mainly depends on their capacity to interact with oncogenic pathways. These pathways, along with other pro-survival functions, may increase mutp53 stabilization, a prerogative for its oncogenic function [1]. Such interplay occurs, for example, between p53-R273H and mammalian target of rapamycin (mTOR) which engages a positive regulatory circuit where mutp53 activates mTOR and this pathway in turn sustains mutp53 expression level by inhibiting autophagy and thus preventing its degradation through this route [2]. Another pathway that may be activated by p53 mutant proteins including p53-R273H is signal transducer and activator of transcription 3 (STAT3) which may increase mutp53 stability by upregulating heat shock protein 90 (HSP90) and contributing to the activation of the mevalonate pathway [3,4,5]. The pro-survival effects of p53-R273H also depend on its capacity to increase intracellular reactive oxygen species (ROS) [6]. Indeed, ROS may sustain cell survival, for example by activating oncogenic pathways such as phosphatidylinositol 3-kinase/ Protein kinase B/ mammalian target of the rapamycin (PI3K/AKT/mTOR) [7] that also promote the release of pro-inflammatory/immune suppressive cytokines including interleukin 6 (IL-6) and interleukin 10 (IL-10) [8,9]. Promoting the release of pro-inflammatory cytokines is another oncogenic trait of mutp53 that creates an inflammatory/immune suppressive milieu which strongly promotes tumorigenesis [10]. Interestingly, it is known that most of the effects induced by mutp53 proteins are opposite to those mediated by wild type p53 (wtp53). This also occurs with respect to the activation of mevalonate [11,12], STAT3 [3,13], mTOR [14] and pro-survival pathways, that, as mentioned above, play a crucial role in sustaining mutp53 stability. While wtp53 has been reported to reduce ROS levels, i.e., interfering by with nicotinamide adenine dinucleotide phosphate (NADPH) oxidase (NOX) 4 expression in the course of transforming growth factor beta (TGF-β) treatment [15], mutp53, in the same condition, upregulates NOX4 and increases the level of ROS [16]. Both wtp53 and mutp53 may regulate pro-oxidant and antioxidant responses, although wtp53 seems to activate the former rather than the latter [17]. Nuclear factor erythroid 2-related factor (Nrf2), the most important transcription factor regulating the antioxidant response, has been shown to interact with both wtp53 and mutp53. Nrf2 is a double face molecule, as it is able to prevent carcinogenesis but also promote cancer resistance to anti-cancer therapies [18,19]. As a result of these opposite roles in carcinogenesis, Nrf2 can be upregulated by wtp53 for cancer prevention and by mutp53 for cancer survival, particularly in the course of anti-cancer therapy. Another process involved in ROS regulation that may be contrarily regulated by mutp53 and wtp53 is autophagy, a catabolic route essential for cellular homeostasis. Indeed, mutp53 is known to reduce autophagy, while wtp53 in most of cases induces it, although its role may depend on its intracellular localization [20]. Besides bulk autophagy, mitophagy plays a pivotal role in restraining ROS increases, even if how mutp53 and wtp53 affect this specific process is still under investigation.

In this study, we performed p53-R273H silencing and overexpression experiments to investigate the role of this mutp53 on cell survival and correlated it with the level of intracellular ROS, cytokine production, mTOR activation, autophagy, mitophagy and the expression of uncoupling protein 2 (UCP2). p53-R273H overexpression was induced in p53 null, p53 knock out (K/O) and wtp53 cancer cells. Interestingly, in the latter cells, p53-R273H led to the activation of wtp53/p21 which prevented all pro-tumorigenic effects induced by this mutp53. This could explain why cancers harboring heterozygous mutp53 are less aggressive than those carrying homozygous mutp53. 

## 2. Material and Methods

### 2.1. Cell Culture and Treatments

Panc1 (human pancreatic cancer cell line, mutp53), ASPC-1 (human pancreatic cancer cell line, p53null) and U87 (human glioblastoma cell line, wtp53) cell lines were grown in RPMI 1640 medium (Thermo Fisher Scientific, Waltham, MA, USA), while HCT116 (human colon cancer cell line, wtp53) and HCT116 p53-/- (human colon cancer cell line, p53 K/O) were maintained in DMEM 1640 medium (Thermo Fisher Scientific, Waltham, MA, USA), both supplemented with 10% fetal bovine serum (FBS) (Corning, NY, USA), L-glutamine, streptomycin (100 μg/mL) (Corning, NY, USA), and penicillin (100 U/mL) (Corning, NY, USA) in 5% CO_2_ at 37 °C. Cells were always detached using Trypsin–EDTA solution (Biological Industries, Cromwell, CT, USA).

In some experiments, Panc1 cells were plated in 6-well plates at a density of 2 × 10^5^ cells/well in 2 mL. The following day, cells were treated with N-acetylcysteine (NAC) (5 mM; Sigma Aldrich, St Louis, MO, USA) for 24 h. Untreated cells were used as controls (CT).

### 2.2. p53 Silencing, mutp53 and wtp53 Transfection

Panc1 cells were seeded into 6-well plates at a density of 2 × 10^5^ cells/well and transfected with empty vector (p-Super) or sip53 plasmid (p-Super-p53) [21] for p53 knockdown the following day using Lipofectamine 2000 (Invitrogen, Waltham, MA, USA) according to the manufacturer’s instructions. After 48 h, a Trypan blue assay was performed, and cells were recovered for further analysis.

ASPC-1, U87, HCT116 wt and HCT116 p53-/- cells were seeded into 6-well plates at a density of 2 × 10^5^ cells/well and were transfected with pcDNA3- p53R273H vector (kindly provided by Prof. D’Orazi) or with empty vector (EV) the following day using Lipofectamine 2000 (Invitrogen) according to the manufacturer’s instructions. In some experiments, HCT116 p53-/- cells were co-transfected with pcDNA3- p53R273H vector and pcDNA3 p53 wt vector (kindly provided by Prof. D’Orazi) or with empty vector (EV) using Lipofectamine 2000 (Invitrogen). After 24 h, a Trypan blue assay was performed and cells were recovered for further analysis. In some other experiments, HCT116 wtp53 cells were pre-treated with pifithrin-α (P) (30 μM, Sigma) or UC2288-p21 inhibitor (p21 inh) (5 μM, Millipore, Burlington, MA, USA). After 24 h, a Trypan blue assay was performed and cells were recovered for further analysis.

### 2.3. Cell Viability

Cell viability was evaluated using a Trypan blue (Sigma Aldrich) exclusion assay. Cells were counted by light microscopy using a Neubauer hemocytometer. The experiments were performed in triplicate and repeated at least three times.

### 2.4. Measurement of Intracellular Reactive Oxygen Species (ROS) Production

To measure reactive oxygen species (ROS) production, 10 μM 2,7-dichlorofluorescein diacetate (DCFDA; Sigma-Aldrich D6883) was added to cell cultures for 15 min and live cells, gated according to their forward scatter (FSC) and side scatter (SSC) properties, were analyzed on a FACScalibur flow cytometer (BD Transduction Laboratories, Franklin Lakes, NJ, USA ) using CELLQuest Pro software (version 6.0, BD Biosciences, Franklin Lakes, NJ, USA). For each analysis, 10,000 events were recorded.

### 2.5. Western Blot Analysis

Following transfections and treatments, cells were washed in 1× PBS, lysed in Radio-Immunoprecipitation Assay (RIPA)buffer (150 mM NaCl, 1% NP-40, 50 mM Tris-HCl (pH 8), 0.5% deoxycholic acid, 0.1% SDS, protease and phosphatase inhibitors) and centrifuged at 14,000 rpm for 20 min at 4 °C. The protein concentration was measured using the Bio-Rad Protein Assay (BIO-RAD laboratories GmbH, Hercules, CA, USA) and 12 μg of protein were subjected to electrophoresis on 4–12% NuPage Bis–Tris gels (Life Technologies, Carlsbad, CA, USA) according to the manufacturer’s instructions. Then, the gels were transferred onto nitrocellulose membranes (BIO-RAD, Hercules, CA, USA) for 2 h in Tris–Glycine buffer. The membranes were blocked in 1× PBS + 0.1% Tween 20 solution containing 3% bovine serum albumin BSA (Serva, Reno, NV, USA), probed with specific antibodies (see Section 2.6 below) and developed using Electrochemiluminescence ECL Blotting Substrate (Advansta, San Josè, CA, USA).

### 2.6. Antibodies

To evaluate the expression of proteins in Western blot analysis, we used the following antibodies: mouse monoclonal anti-p53 (1:100) (clone DO-1, Santa Cruz Biotechnology Inc., sc-126, Heidelberg, Germany), rabbit monoclonal anti-phospho-4E-BP1 (Thr37/46) (236B4) (1:1000) (Cell Signaling, 2855, Danvers, MA, USA), rabbit monoclonal anti-4E-BP1 (1:2000) (Proteintech, 60246-1-Ig, Rosemont, IL, USA), mouse monoclonal anti-p62 (SQSMT1) (1:1000) (BD Transduction Laboratories, 610832, Franklin Lakes, NJ, USA), mouse monoclonal anti-p21 (1:100) (clone F-8, Santa Cruz Biotechnology Inc., sc-271610), mouse monoclonal anti-cyclin D1 (1:100) (clone A-12, Santa Cruz Biotechnology Inc., sc-8396), mouse monoclonal anti-HADHA (1:100) (clone F-8, Santa Cruz Biotechnology Inc., sc-374497), mouse monoclonal anti-UCP2 (1:100) (clone G-6, Santa Cruz Biotechnology Inc., sc-390189), mouse monoclonal anti-Nrf2 (1:100) (clone A-10, Santa Cruz Biotechnology Inc., sc-365949), and mouse monoclonal anti-NQO1 (clone H-9, Santa Cruz Biotechnology Inc., sc-376023). Mouse monoclonal anti-β-actin (1:10,000) (Sigma Aldrich) was used as a loading control. Goat anti-mouse IgG-HRP (1:30,000) (Bethyl Laboratories, A90-116P) ad goat anti-rabbit IgG-HRP (1:30,000) (Bethyl Laboratories, A120-101P, Montgomery, TX, USA) were used as secondary antibodies. All primary and secondary antibodies were diluted in PBS + 0.1% Tween20 solution containing 3% BSA (SERVA, Reno, NV, USA).

### 2.7. Chemiluminescent Immunometric Assay

After culturing for 24 h, supernatants derived from transfections were analyzed using a magnetic Luminex assay using a human pre-mixed multi-analyte kit (R&D Systems Bio-Techne, Minneapolis, MN, USA) according to the manufacturer’s instructions. 

### 2.8. Indirect Immunofluorescence Assay (IFA)

An indirect immunofluorescence assay was used to analyze HADHA in p53wt and p53-/- HCT116 cells 24 h transfected with mutp53-R273H. Cells were applied onto multispot microscope slides and air-dried, then fixed with 2% paraformaldehyde (Electron Microscopy Science, 157-8, Hatfield, PA, USA) for 30 min and permeabilized with 0.1% Triton X-100 (Sigma Aldrich, T-8787) for 5 min. Cells were then incubated with a primary monoclonal antibody against HADHA (clone F-8, Santa Cruz Biotechnology Inc., sc-374497) for 1 h at room temperature, washed with PBS and incubated with a polyclonal conjugated-Cy3 sheep anti-mouse antibody (Jackson ImmunoResearch, 515-165-062, Ely, UK) for 30 min. Cells were also stained with 1 μg/mL 4′,6′-diamidino-2-phenylindole (DAPI) (Sigma-Aldrich), mounted with glycerol:PBS (1:1) and observed on a fluorescence microscope (Olympus BX53, Norfolk, VA, USA).

### 2.9. Statistical Analysis

Results are represented as the mean ± standard deviation (SD) of at least three independent experiments and a two-tailed Student’s t-test was used to demonstrate statistical significance. Differences were considered statistically significant when *p*-values were at least <0.05. 

## 3. Results

### 3.1. p53-R273H Silencing Reduces Cell Survival, Intracellular ROS, Cytokine Release and mTOR Activation While Inducing Autophagy in Panc1 Cells

**Figure 1:**
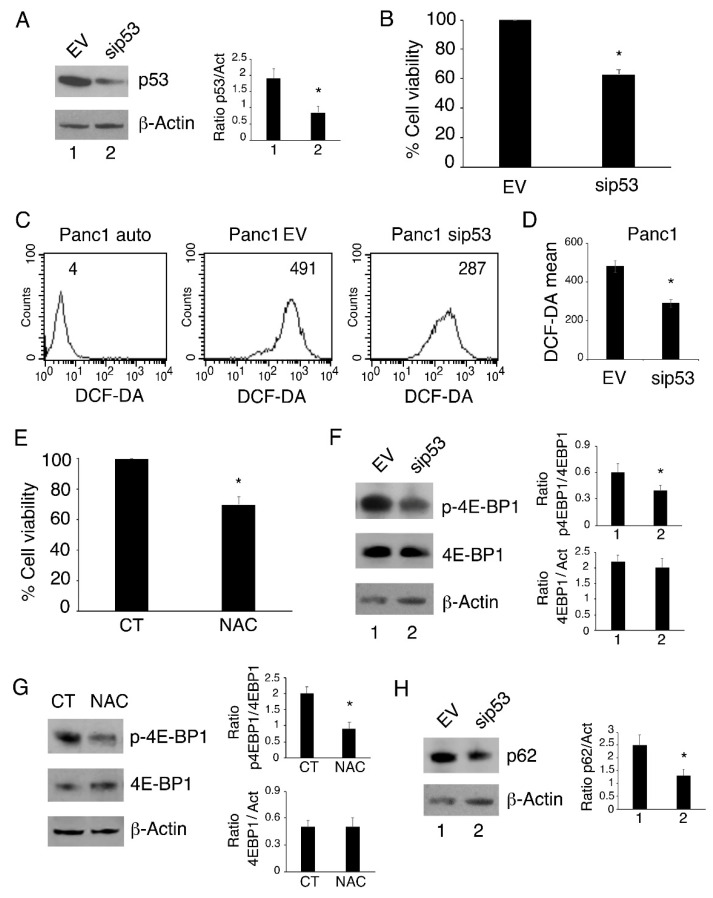
p53-R273H silencing reduces cell survival and intracellular reactive oxygen species (ROS) and induces autophagy in Panc1 cells. Panc1 cells were transfected with empty vector (EV, 1) or the sip53 vector (sip53, 2) and after 48 h (**A**) p53 expression was evaluated by Western blot analysis. Actin was used as loading control; the histograms represent the mean + standard deviation (SD) of the densitometric analysis of the ratio of p53 to actin. (**B**) Cell survival was evaluated by Trypan blue exclusion assay. The histograms represent the percentage of cell viability relative to the control; data are represented as the mean + SD of more than 3 experiments. * *p* value < 0.05. (**C**) Intracellular ROS level was measured by FACS analysis using DCF-DA. Mean fluorescence intensity (MFI) is indicated. One representative experiment out of three is reported. Auto = autofluorescence. (**D**) Histograms represent the mean of the MFI of DCF-DA + SD. * *p* value < 0.05. (**E**) The survival of Panc1 cells treated with n-acetylcysteine (NAC) (5mM) for 24 h was evaluated by Trypan blue exclusion. The histograms represent the percentage of cell viability relative to the control; data are represented as the mean + SD of more than 3 experiments. * *p* value < 0.05. (**F**) The expression of p-4E-BP1 and 4-E-BP1 were analyzed by Western blot analysis. Actin was used as the loading control. The histograms represent the mean + SD of the densitometric analysis of the ratio between the protein and the appropriate control. * *p* value < 0.05. (**G**) Protein expression of p-4E-BP1 and 4-E-BP1 in NAC-treated or untreated cells were analyzed by Western blot. Actin was used as the loading control. The histograms represent the mean + SD of the densitometric analysis of the ratio between the protein and the appropriate control. * *p* value < 0.05. (**H**) The expression of p62 in Panc1 cells transfected with empty vector (EV, 1) or the sip53 vector (sip53, 2) was analyzed by Western blot. Actin was used as the loading control. The histograms represent the mean + SD of the densitometric analysis of the ratio between the protein and the appropriate control. * *p* value < 0.05.

We evaluated the role of mutp53 on cell survival by silencing it using a specific siRNA in Panc1 cells, a pancreatic cell line harboring the *p53-R273H* mutation. We found that p53-R273H silencing reduced cell survival in comparison to the scramble treatment (Figure 1A,B). We also found that this effect was correlated with a reduction in intracellular ROS (Figure 1C,D), whose role in cell survival has been previously reported [6]. Here, we confirmed these findings by using n-acetylcysteine (NAC), a ROS scavenger, that indeed impaired Panc1 cell survival similarly to mutp53 silencing (Figure 1E). As mTOR is one of the oncogenic pathways activated by mutp53 [2], and given that ROS contribute to the activation of this oncogenic pathway [7], we then evaluated the phosphorylation status of 4E-BP1, an mTOR target in Panc1 cells *undergoing mutp53 silencing* or NAC treatment. As shown in Figure 1F,G, both treatments reduced the activation of 4E-BP1, suggesting that ROS could play a role in mTOR activation induced by p53-R273H. mTOR is the master regulator of autophagy and its activation by mutp53 has been reported to prevent its degradation through autophagy [22]. Therefore, we also evaluated autophagic flux in scramble and mutp53 silenced Panc1 cells by assessing the expression level of p62/sequestosome 1 (SQSTM1), a molecule that is mainly degraded through autophagy. As shown in Figure 1H, the expression level of p62/SQSTM1 was reduced in mutp53-silenced Panc1 cells, suggesting that its knockdown removed the block on autophagy. Altogether, these results suggest that p53-R273H silencing reduces cells survival, ROS and mTOR activation while restoring autophagy in Panc1 cells.

### 3.2. mutp53-R273H Overexpression Increases Cell Survival, ROS and Inflammatory Cytokine Release in p53 Null Cells; Effects Were Not Observed in wtp53 Cells

**Figure 2:**
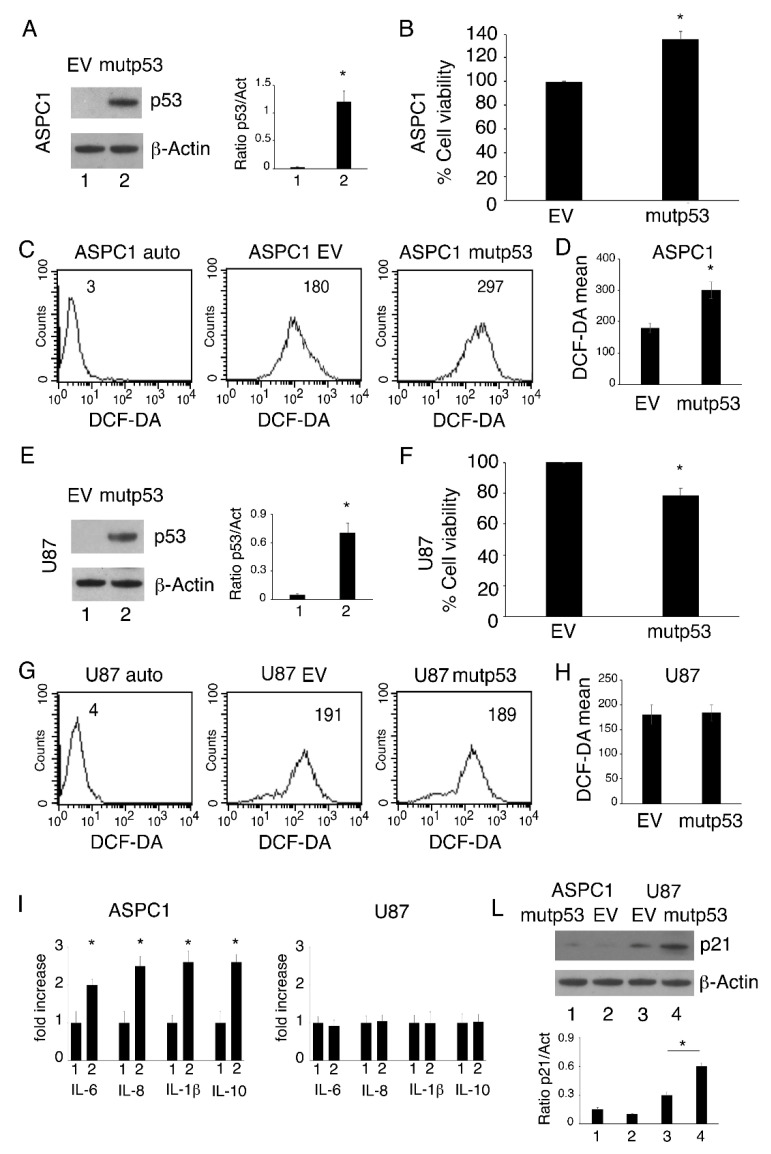
p53-R273H overexpression increases cell survival, ROS, and inflammatory cytokine release in ASPC1 p53-null cells and U87 wtp53-carrying cells. ASPC1 and U87 cell lines were transfected with empty vector (EV,1) or the pcDNA3- p53R273H vector (mutp53, 2). After 24 h: (**A**) and (**E**) transfection was evaluated by Western blot analysis of the expression of p53. The histograms represent the mean + SD of the densitometric analysis of the ratio of p53 to actin. * *p* value < 0.05. (**B**) and (**F**) Cell survival was evaluated by Trypan blue exclusion assay. The histograms represent the percentage of cell viability relative to the appropriate control; data are represented as the mean + SD of more than 3 experiments. * *p* value < 0.05. (**C**) and (**G**) Intracellular ROS level was measured by FACS analysis using DCF-DA to stain cells. Mean fluorescence intensity (MFI) is indicated. One representative experiment out of three is reported. (**D**) and (**H**) Histograms represent the mean of the MFI of DCF-DA plus SD. * *p* value < 0.05. (**I**) After 24 h of culture, supernatants derived from cells transfected with EV or mutp53 were collected and IL-6, IL-8, IL-1β and IL-10 were measured by Luminex assay. Histograms represent the fold increase of the values for each cytokine compared to EV transfection. The results were considered significant (*) when the *p* value was < 0.05. (**L**) p21 expression was evaluated by Western blot analysis. Actin was used as the loading control. The histograms represent the mean + SD of the densitometric analysis of the ratio of p21 to actin. * *p* value < 0.05.

We then investigated if the transfection of p53-R273H into cancer cells that do not carry this mutation could induce opposite effects compared to p53-R273H silencing. *We found* that p53-R273H overexpression (Figure 2A) promoted cell survival (Figure 2B) and increased intracellular ROS (Figure 2C,D) in pancreatic cancer ASPC1 p53-null cells. Interestingly, the overexpression of mutp53 in U87 (Figure 2E), a glioblastoma cell line carrying wtp53, slightly affected cell survival (Figure 2F) and did not increase intracellular ROS (Figure 2G,H). As ROS are strongly inter-connected with the release of pro-inflammatory cytokines and other molecules involved in pro-tumorigenic effects induced by mutp53 [10], we then evaluated inflammatory cytokine release by ASPC1 and U87 cells transfected with p53-R273H or with empty vector (EV). Interestingly, multi-analyte Luminex assay analysis indicated that mutp53 increased the release of pro-inflammatory/immune suppressive cytokines by ASPC1 cells and, once again, this effect was not observed in U87 cells (Figure 2I). Finally, we found that p53-R273H overexpression upregulated the expression level of p21 in U87 cells, while it only slightly affected it in ASPC1 cells (Figure 2L), suggesting that a reactivation of wtp53 by p53-R273H transfection could counteract the pro-survival effects induced by mutp53.

### 3.3. p53-R273H Overexpression Increases Cell Survival, ROS and Inflammatory Cytokine Release in HCT116 p53-/- But Not in HCT116 wtp53 Cells

**Figure 3:**
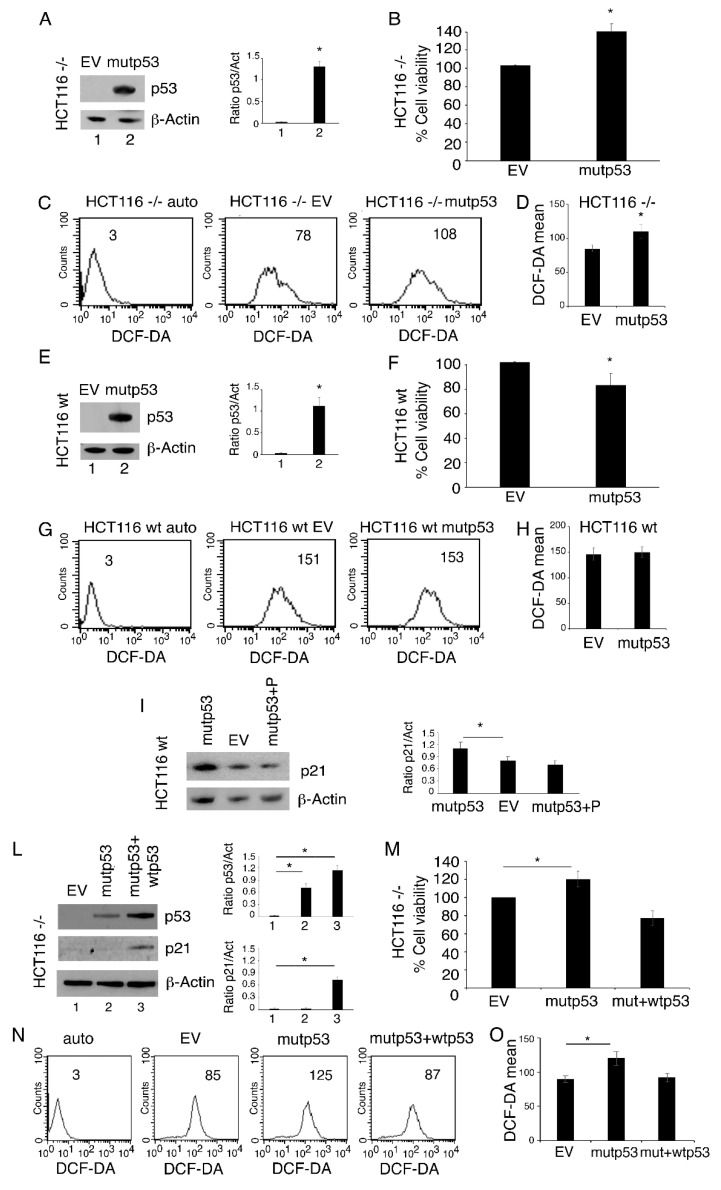
*p53-R273H* overexpression increases cell survival and ROS in HCT116 p53-/- but not in HCT116 wtp53 cells. HCT116 p53-/- and HCT116 wtp53 cells were transfected with empty vector (EV, 1) or pcDNA3- p53R273H vector (mutp53, 2). In some experiments, HCT116 p53-/- cells were co-transfected with the pcDNA3- p53R273H vector and the pcDNA3 p53wt vector (mutp53 + wtp53, 3). (**A**) and (**E**) After 24 h, transfection was evaluated by Western blot analysis to examine the expression of p53. The histograms represent the mean + SD of the densitometric analysis of the ratio of p53 to actin. * *p* value < 0.05. (**B**), (**F**) and (**M**) Cell survival was evaluated by Trypan blue exclusion assay. The histograms represent the percentage of cell viability relative to the appropriate control; data are represented as the mean + SD of more than 3 experiments. The results were considered significant (*) when *p* value < 0.05. (**C**), (**G**) and (**N**) Intracellular ROS level was measured by FACS analysis using DCF-DA to stain cells. Mean fluorescence intensity (MFI) is indicated. One representative experiment out of three is reported. (**D**), (**H**) and (**O**) Histograms represent the mean of the MFI of DCF-DA + SD. * *p* value < 0.05. (**I**) HCT116 wtp53 cells were transfected with empty vector (EV) or pre-treated with pifihtrin-α (P) (30 μM) and then transfected with pcDNA3- p53R273H (mutp53) to evaluate p21 expression by Western blot analysis. Controls were untreated. The histograms represent the mean + SD of the densitometric analysis of the ratio of p21 to actin. * *p* value < 0.05. (**L**) The expression of p53 and p21 was evaluated by Western blot analysis of co-transfected HCT116 p53-/- (mutp53 + wtp53). The histograms represent the mean + SD of the densitometric analysis of the ratio of p53 or p21 to actin. * *p* value < 0.05.

**Figure 4:**
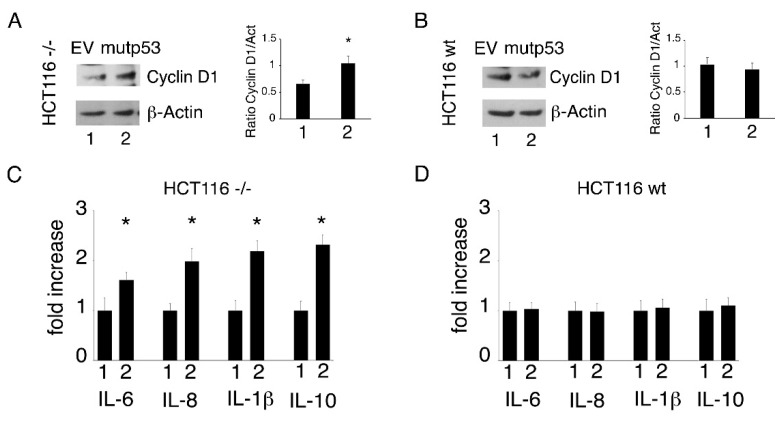
*p53-R273H* overexpression differently modulates cyclin D1 expression in HCT116 p53-/- and HCT116 wtp53 cells and increases inflammatory cytokine release in HCT116 p53-/- but not in HCT116 wtp53 cells. HCT116 p53-/- and HCT116 wtp53 cells were transfected with empty vector (EV, 1) or the pcDNA3- p53R273H vector (mutp53, 2). (**A**) and (**B**) After a 24 h transfection, the expression of cyclin D1 was evaluated by Western blot analysis. The histograms represent the mean + SD of the densitometric analysis of the ratio of cyclin D1 to Actin. * *p* value < 0.05. (**C**) and (**D**) After 24 h of culture, supernatants derived from HCT116 p53-/- and HCT116 wtp53 cells transfected with EV or mutp53 were collected, and IL-6, IL-8, IL-1β and IL-10 were measured by Luminex assay. Histograms represent the fold increase of the values of each cytokine compared to EV transfected cells. The results were considered significant (*) when the *p*-value was < 0.05.

To investigate the effect of p53-R273H transfection in another cell type and to exclude that the different outcomes observed in p53-null and wtp53-carrying cells (ASPC and U87, respectively) could be due to the different cellular background, we repeated the p53-R273H transfection experiments in HCT116 cells carrying wtp53 and in the same cells with p53 knocked out. We found that p53-R273H transfection (Figure 3A) increased cell survival (Figure 3B) and ROS levels (Figure 3C,D) in HCT116 p53-/- cells and that it did not induce such an effect in HCT116 wtp53 cells (Figure 3E–H) in which, similarly to what was observed in U87 cells, p21 was upregulated (Figure 3I). The role of wtp53 activation in p21 upregulation was then demonstrated by treating HCT116 wtp53 cells with pifithrin-α, a p53 inhibitor, which prevented p21 upregulation following *p53-R273H* transfection (Figure 3I). Next, to further demonstrate the capacity of wtp53 to counteract the effects induced by mutp53, we co-transfected wtp53 and mutp53 into HCT116 p53-/- cells (Figure 3L) and found that p21 was upregulated but cell survival and ROS did not increase (Figure 3M–O). To investigate the mechanism through which the activation of the p53/p21 axis could counteract the pro-survival effect induced by mutp53, we evaluated the expression of cyclin D1 which can be activated by mutp53 and repressed by wtp53 [23,24]. As shown in Figure 4A,B, cyclin D1 expression increased in HCT116 p53-/- cells while it slightly decreased in wtp53 cells; thus, this may be one of the reasons why only the p53-/- cells showed an increase in cell proliferation following mutp53 transfection. Finally, we found that in HCT116 p53-/-cells, *p53-R273H* promoted the release of pro-inflammatory/immune suppressive cytokines, and again, such an effect was not observed in HCT116 wtp53 cells (Figure 4C,D). Altogether, these results suggest that the activation of wtp53 also prevents several pro-survival effects induced by p53-R273H in cells with a similar background.

### 3.4. p53-R273H Overexpression in HCT116 p53-/- Cells Induces mTOR Activation, Reduces Autophagy and Mitophagy and Downregulates UCP2 Expression

**Figure 5:**
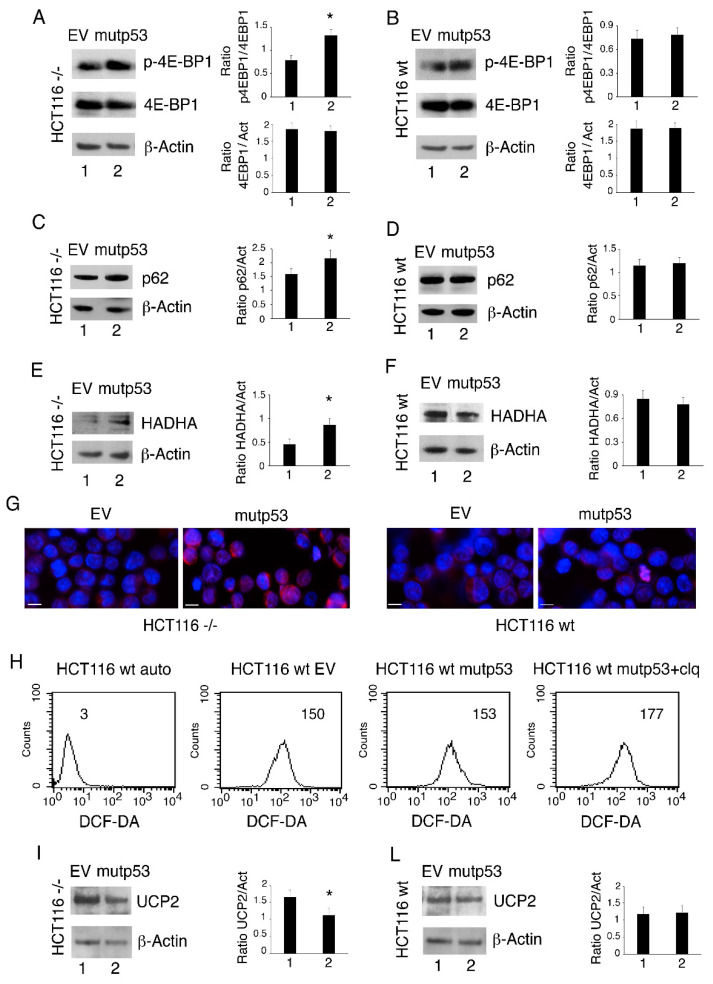
*p53-R273H* transfection in HCT116 p53-/- cells induces mammalian target of rapamycin (mTOR) activation, reduces autophagy and mitophagy, and downregulates mitochondrial uncoupling proteins 2 (UCP2) expression. HCT116 wtp53 and HCT116 p53-/- cells were transfected with empty vector (EV, 1) or the pcDNA3- p53R273H vector (mutp53, 2). (**A**) and (**B**) After 24 h, the expression of p-4E-BP1 and 4E-BP1 was analyzed by Western blot. Actin was used as the loading control. The histograms represent the mean + SD of the densitometric analysis of the ratio between the protein and the appropriate control. * *p* value < 0.05. (**C**) and (**D**) p62 expression and (**E**) and (**F**) HADHA expression as evaluated by Western blot analysis. The histograms represent the mean + SD of the densitometric analysis of the ratio between the protein and the appropriate control. * *p* value < 0.05. (**G**) HADHA expression in mutp53-R273H-transfected HCT116 p53-/- and HCT116 wtp53 cells as evaluated by immunofluorescence assay (IFA) after 24 h. Nuclei are stained in blue with DAPI and HADHA in red. Scale bar: 10 μm. (**H**) Intracellular ROS level was measured by FACS analysis using DCF-DA to stain cells. Mean fluorescence intensity (MFI) is indicated. One representative experiment out of three is reported. (**I**) and (**L**) UCP2 expression was evaluated by Western blot analysis. Actin was used as the loading control. The histograms represent the mean + SD of the densitometric analysis of the ratio between the protein and the appropriate control. * *p* value < 0.05.

As ROS contribute to the activation of oncogenic pathways such as mTOR [7], the phosphorylation of 4-E-BP1, an mTOR target, was then investigated in HCT116 p53-/- and wtp53 cells following p53-R273H transfection. We found that p-4E-BP1 increased in both HCT116 p53-/- cells and HCT116 wtp53 cells (Figure 5A,B). As mTOR is a negative regulator of autophagy, we also found that p62/SQSTM1 accumulated in HCT116 p53-/- cells, suggesting a reduction in autophagic flux (Figure 5C,D).

Besides bulk autophagy, a selective form of autophagy known as mitophagy plays a major role in the elimination of damaged mitochondria, which are the main source of ROS. As ROS increased following p53-R273H transfection into cancer cells lacking wtp53, we then evaluated mitophagy in HCT116 p53-/- or mutp53-transfected wtp53 cells. The results shown in Figure 5E–G indicate that the expression level of HADHA, a molecule degraded through mitophagy, increased following p53-R273H transfection in HCT116 p53-/- cells and was slightly affected in HCT116 wtp53 cells as evaluated by Western blot and IFA, respectively. This suggests that like autophagy, mitophagy was also impaired by p53-R273H transfection into cells lacking wtp53 and could further contribute to the observed ROS increase. To investigate the role of autophagy/mitophagy inhibition in ROS increase, we used chloroquine, an inhibitor of the final steps in the autophagy pathway, in HCT116 wtp53 cells and found that chloroquine treatment also increased ROS in these cells (Figure 5H). Interestingly, mitophagy is also under the control of mTOR, as we recently observed in Kaposi’s Sarcoma-associated Herpesvirus (KSHV)-infected Human umbilical vein endothelial cells (HUVECs) cells [25]. One of the proteins playing a key role in ROS production and known to be upregulated by mutp53 is UCP2 [26]; therefore, we next evaluated the impact of p53-R273H transfection on this protein. As shown in Figure 5I,L, UCP2 expression decreased in HCT116 p53-/- cells while it was slightly affected in HCT116 wtp53 cells. Interestingly, UCP2 expression has been correlated with AMP-activated protein kinase (AMPK) [26] and mTOR activation [27]; accordingly, in this study, we observed that UCP2 was downregulated in HCT116 p53-/-cells in which mTOR activity increased. Altogether, these results indicate that autophagy/mitophagy reduction and UCP2 downregulation could be responsible for the observed ROS increases in HCT116 p53-/- cells transfected with mutp53.

### 3.5. The Nrf2 Antioxidant Program is Activated in HCT116 p53-/- But Not in HCT116 wtp53 Following p53-R273H Transfection

**Figure 6:**
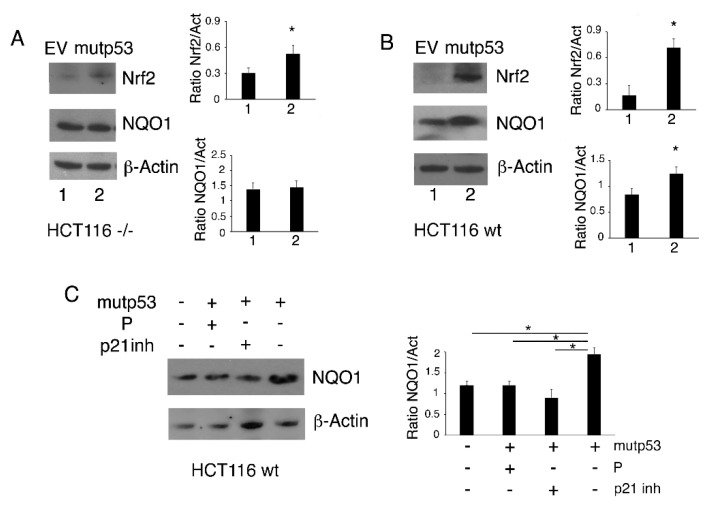
The Nrf2/NQO1 axis is activated in HCT116 p53-/- cells but not in HCT116 wtp53 cells. HCT116 p53-/- and HCT116 wtp53 cells were transfected with empty vector (EV, 1) or the pcDNA3- p53R273H vector (mutp53, 2). (**A**) and (**B**) After 24 h, Nrf2 and NQO1 expression were evaluated by Western blot analysis. Actin was used as the loading control. The histograms represent the mean + SD of the densitometric analysis of the ratio between Nrf2 or NQO1 and actin. * *p* value < 0.05. (**C**) NQO1 expression was evaluated by Western blot in HCT116 wtp53 cells pre-treated with pifithrin-α (P) (30 μM) or UC2288-p21 inhibitor (p21 inh) (5 μM) and then transfected as described above. Control cells were untreated. The histograms represent the mean + SD of the densitometric analysis of the ratio between NQO1 and actin. * *p* value < 0.05.

ROS level is strongly regulated by the antioxidant response, whose main activator is Nrf2. Therefore, we also evaluated the expression of this transcription factor in HCT116 p53-/- cells and HCT116 wtp53 cells following p53-R273H transfection. As shown in Figure 6A,B, Nrf2 was upregulated by p53-R273H transfection in both cell lines, independently of the presence of wtp53. As mutp53 has been reported to modulate the transcriptional program of Nrf2 [28], we then evaluated the expression of NAD(P)H dehydrogenase quinone 1 (NQO1), an Nrf2 target molecule whose expression has been shown to be reduced by mutp53 [29]. We found that the expression of NQO1 was slightly affected in HCT116 p53-/- cells transfected with p53-R273H, while it more clearly increased in HCT116 wtp53 cells (Figure 6A). According to previous findings, NQO1 may engage in a positive feedback loop with wtp53 [18].

Finally, to evaluate the role of the wtp53/21 axis in NQO1 upregulation, we used the p53 inhibitor pifithrin-α or the p21 inhibitor UC2288 in HCT116 wtp53 cells following *p53-R273H* transfection and found that both treatments counteracted the increase in NQO1 expression induced by *p53-R273H transfection (Figure 6C)*.

Altogether, these results suggest that the antioxidant response activated by the wtp53/p21 axis prevented the ROS increase induced by p53-R273H expression in cells carrying wtp53.

## 4. Discussion

In this study, we found that p53-R273H promoted cell survival by increasing ROS and promoting cytokine release in cancer cells lacking wtp53, and these effects were counteracted by the presence of wtp53. This could explain why cancers carrying homozygous mutp53 display more aggressive behaviors and why cancer cells tend to lose the wtp53 allele during malignant progression [30]. We found that the differences in intracellular ROS level observed in p53-/- HCT116 cells and wtp53-carrying HCT116 cells following p53-R273H transfection correlated with the downregulation of UCP2 expression level in cancer cells lacking wtp53. UCP2 is a protein that can help to dissipate the proton gradient and prevent the proton-motive force from becoming excessive, reducing the ROS produced by electron transport [31]. Interestingly, UCP2 has been also reported to help to preserve mitochondrial integrity by preventing the loss of membrane potential [32], and thus its downregulation by p53-R273H could increase mitochondrial damage and ROS production.

The downregulation of the AMPK/PGC-1α/UCP2 axis has previously been shown to strongly contribute to mutp53 ROS increase and the subsequently induced oncogenesis [26]. Interestingly, here we found that UCP2 was downregulated following p53-R273H-transfection only in cancer cells lacking wtp53 in which mTOR activation was also observed.

Another important finding of this study was that to further increase ROS, p53-R273H transfection reduced both autophagy and mitophagy in these cells. The dysregulation of these processes plays an important role in cancer, although autophagy may play different roles depending on the stage of carcinogenesis [33]. The reduction of mitophagy by mutp53 transfection in cells lacking wtp53 represents a new mechanism through which p53-R273H could promote ROS production and sustain cell survival.

The antioxidant response, mainly regulated by the transcription factor Nrf2, plays a key role in the control of ROS. We found that it was upregulated following p53-R273H transfection independently of p53 status. However, according to a study showing that mutp53 can tune Nrf2 transcription activity, resulting in the upregulation of specific targets [28], here we found that NQO1, antioxidant molecular target of Nrf2, was upregulated only in cancer cells carrying wtp53.

In cells lacking wtp53, Nrf2 upregulation could be due to the positive feedback loop induced with mutp53 [34], while in the case of cancer cells carrying wtp53, p53-R273H transfection could lead to the upregulation of Nrf2 through the activation of the wtp53/p21 axis. Indeed, in response to oxidative stress, p21 may interact with Nrf2 and compete with Kelch-like ECH-associated protein 1 (Keap1) for binding to this transcription factor, preventing its degradation and thus increasing its stabilization [35]. In agreement with these findings, here we observed that p53-R273H transfection upregulated p21 in wtp53-carrying cells, and that its inhibition by UC2288 as well as the inhibition of wtp53 by pifithrin-α prevented NQO1 upregulation. Although cancer cells also upregulate the antioxidant response to avoid increases so high that they could cause cell death, ROS increases may promote tumorigenesis by several means, particularly due to their interplay with the inflammatory cytokines and oncogenic pathways that regulate their release [36,37]. These cytokines may, in turn, increase ROS production in a positive regulatory circuit that amplifies pro-tumorigenic inflammation [37,38]. Oxidative stress may promote carcinogenesis by several other mechanisms, i.e., by favoring DNA damage and negatively modulating the DNA damage repair response (DDR) [39]. The DDR has been shown to be reduced by mutp53 through the impairment of ataxia-telangiectasia mutated (ATM) activation [40]; therefore, it will be interesting to investigate if its mediated increase in ROS could contribute to such an effect. According to the findings that conversely to mutp53, wtp53 may inhibit the activation of several pro-survival pathways and counteract inflammatory cytokine release, in this study, we found that in wtp53-carrying cancer cells, the activation of p21 following p53-R273H transfection counteracted cell proliferation, ROS production and cytokine release. This suggests that despite the dominant negative effect exerted by mutp53 on wtp53, the activation of the latter may still restrain all pro-tumorigenic effects that we found in this study to be induced by p53-R273H.

In conclusion, this study highlights that *ROS increase (due to several mechanisms including the dysregulation of bulk autophagy, mitophagy and UCP2 downregulation) plays a key role in* the pro-survival effect induced by p53-*R273H,* in correlation with *m-TOR activation and pro-inflammatory/immune suppressive cytokine release. This study also unveils the capacity of wtp53 to restrain these pro-tumorigenic effects induced by* p53-*R273H transfection*.

## Data Availability

The datasets generated and/or analyzed during the current study are available from the corresponding author upon reasonable request.
